# P33. NK-92 cellular immunotherapy as an alternative to donor derived peripheral blood NK cells

**DOI:** 10.1186/2051-1426-2-S2-P24

**Published:** 2014-03-12

**Authors:** H Klingemann

**Affiliations:** 1 Conkwest, Cambridge, MA, USA

## Introduction

Infusions with cytokine-activated NK cells obtained from blood of MHC mismatched donors have shown some promising results especially in patients with myeloid leukaemia. There is also indirect evidence that infusions of NK cells as part of a stem cell transplant result in lower relapse rates post-transplant when donor and recipient are mismatched for KIR receptors on NK cells.

## Methods

Collecting NK cells form donors requires them to undergo leukapheresis with subsequent removal of CD3+ lymphocytes (to prevent GvHD) and expansion and activation of the NK cell enriched fraction with IL-2. In contrast, the continuously growing NK cell line NK-92 can be easily expanded to clinical scale in bioreactors. The broad cytotoxicity of NK-92 is due to the lack of most of the KIR receptors while expressing a range of activating receptors.

## Results

We report here results from concluded and ongoing clinical phase I studies with NK-92 cells in patients with advanced cancer, that confirm its safety profile. Anti-tumour responses were seen in patients with some advanced haematological malignancies and solid tumours. NK-92 also provides a platform for further genetical engineering. A variant has been generated that expresses a high affinity FcgIIIRA receptor that can augment the treatment efficacy of mAbs that utilise ADCC to kill target cells. Various investigators are using the NK-92 cells as a platform to introduce specific tumour antigen receptors (i.e. CAR) to make them targeted to specific tumors such as melanoma, myeloma, leukaemia or brain cancer. Video-lapse studies show that those CAR engineered NK-92 cells specifically kill tumour antigen expressing cancer cells and are able to do 'serial killing'. The cell expansion process for NK-92 has been streamlined and made more economical using various bioreactor designs.

## Conclusion

The human clinical grade NK-92 cell line has many advantages over peripheral blood NK cells as a cell therapy product for cancer patients. Results from phase I clinical trials confirm its safety profile. This unique cell line is now tested in phase II studies for efficacy in various cancers and is also further developed through genetic engineering to target specific tumours through CAR. NK-92 are an off the shelf tumour targeted local and systemic cell treatment.

